# Nutrition Impact Symptoms Are Prognostic of Quality of Life and Mortality After Surgery for Oesophageal Cancer

**DOI:** 10.3390/cancers10090318

**Published:** 2018-09-07

**Authors:** Poorna Anandavadivelan, Lena Martin, Therese Djärv, Asif Johar, Pernilla Lagergren

**Affiliations:** 1 Surgical Care Sciences, Department of Molecular Medicine and Surgery, Karolinska Institutet, Karolinska University Hospital, 171 76 Stockholm, Sweden; asif.johar@ki.se (A.J.); pernilla.lagergren@ki.se (P.L.); 2 Department of Biosciences and Nutrition, Karolinska Institutet, 171 76 Stockholm, Sweden; lena.martin@ki.se; 3 Function Area Clinical Nutrition, Karolinska University Hospital, 141 86 Stockholm, Sweden; 4 Department of Medicine, Solna, Karolinska Institutet, 171 76 Stockholm, Sweden; therese.djarv@sll.se; 5 Department of Surgery and Cancer, Imperial College London, London SW7 2AZ, UK

**Keywords:** oesophageal neoplasms, quality of life, survival, nutrition impact symptoms, oesophagectomy

## Abstract

We aimed to clarify the influence of nutritional problems after surgery for oesophageal cancer on functional health related quality of life (HRQOL) and survival. A prospective nationwide cohort of oesophageal cancer patients operated 2001–2005 in Sweden with 6 months postoperative follow up was used. Nutritional problems were categorized as low/moderate/severe/very severe based on weight loss and nutrition impact symptoms. An ANCOVA model calculated mean score differences (MD) with 95% confidence intervals (CI) of global quality of life (QOL), social and physical function scores, stratified by preoperative body mass index (BMI) <25 and ≥25. A Cox proportional hazards model produced hazard ratios (HR) with 95% CI for overall 5-year survival. Of 358 patients, 196 (55%) had preoperative BMI ≥25. Very severe and severe nutritional problems were associated with worse HRQOL in both BMI groups. E.g. MD’s for global QOL among ‘very severe’ group was −29 (95% CI −39–−19) and −20 (95% CI −29–−11) for <25 and ≥25 BMI, respectively, compared to the ‘low’ group. Overall 5-year survival among ‘very severe’ and BMI ≥ 25 was worse; HR 4.6 (95% CI 1.4–15.6). Intense nutritional problems negatively impact postoperative HRQOL and combined with preoperative BMI ≥ 25 are associated with poorer 5-year overall survival representing a group needing greater clinical attention.

## 1. Introduction

Oesophageal cancer ranks ninth among the most common cancer types worldwide [1]. The curative treatment includes extensive surgery typically preceded by chemotherapy or chemo radiotherapy. Despite improving survival rates, it is evident that the post-operative survivorship is challenging with decreased health related quality of life (HRQOL), eating difficulties, malnutrition and poor long-term survival [2]. Clinically significant weight loss defined as malnutrition are persistent problems after surgery [3]. Recovery of global quality of life (QOL), physical function and social function at 6 months are prognostic indicators of poor survival [4]. It is imperative to determine factors that are associated with poor HRQOL and survival that are of prognostic value to identify patients at risk and in turn facilitate tailored interventions. This study aimed to clarify if symptoms that impact oral intake, defined as nutrition impact symptoms and clinically significant weight loss can explain deterioration in global quality of life, physical function and social function at 6 months after surgery for oesophageal cancer and the 5-year overall survival, taking into account variations in pre-operative body mass index (BMI). 

## 2. Material and Methods

### 2.1. Data Sources

The data for this study was obtained from a population based cohort of patients who underwent surgical resection for oesophageal cancer in Sweden between April 2001 and December 2005. The study was approved by the ethics committee at Karolinska University Hospital, Karolinska Institute, and the Regional Ethical Review Board, Stockholm, Sweden. The ethical approvals by the Regional Ethical Review Board in Stockholm, Sweden for this study are DNR: 01-064, date: 05/02/2001; DNR: 01-340, date: 28/05/2001; and DNR: 05-1491-32, date: 19/12/2015. In total 175 of 179 (97%) eligible hospitals in Sweden participated in the study. The data collection was centrally coordinated and administered by a project manager. Patients who were operated on were identified with a histopathology report of a confirmed diagnosis of oesophageal or cardia cancer from the pathology units. Thereafter physicians were contacted to obtain informed consent from patients and retrieval of individual data. The medical charts obtained were scrutinized meticulously by a dedicated team of researchers and clinicians who were not involved in the patients’ treatment, based on a detailed and predefined study protocol to ensure uniformity of the clinical data. A total of 616 patients (90% of all surgically treated) were included until the end date of inclusion in December 2005. The 10% non-inclusion was mainly owing to non-participating hospitals. 

Patients included in the cohort were further contacted by mail for informed consent and follow up on HRQOL and body weight measures at 6 months, 3, 5 and 10 years. The 15 year follow up is ongoing. This present study has used data collected at 6 months after surgery. Principally two questionnaires were used to collect data on HRQOL, the general questionnaire QLQ-C30 [5] and the oesophageal specific module QLQ-OES18 [6] from the European Organisation for Research and Treatment of Cancer (EORTC). Both the questionnaires have been validated previously for their use in patients with cancer and also specifically for patients with oesophageal cancer [7,8,9,10,11]. The filled-in questionnaires were sent to a central administration and not to the patient’s physician or hospital department and thus were anonymous. Up to three reminders were sent, if appropriate, for unreturned questionnaires. 

A linkage of the patient’s unique personal identity number to the Swedish Causes of Death Register was made to obtain survival data. The causes of death register contains information on date of death for all Swedish residents since 1952 and has 99.2% completeness for cause-related death [12]. 

### 2.2. Study Design

#### 2.2.1. Exposure 

**Figure 1:**
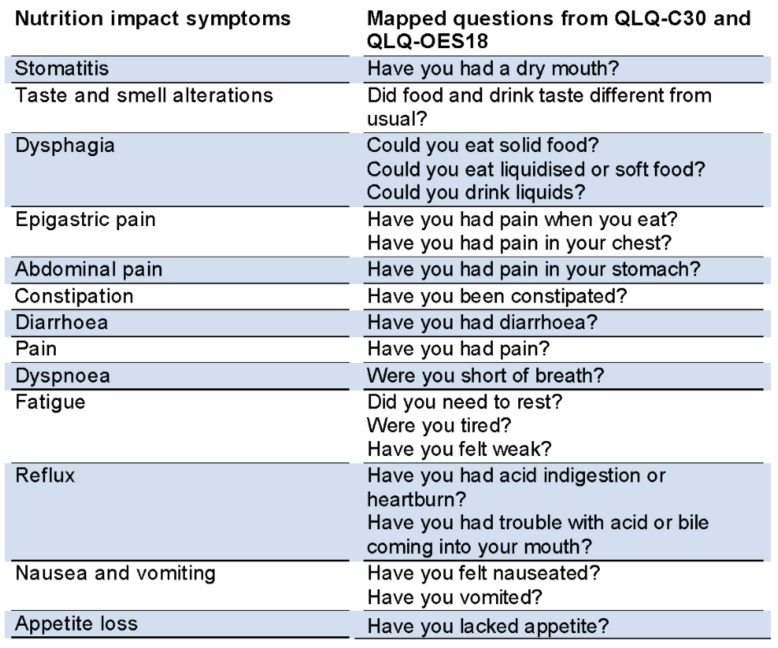
Nutrition impact symptoms mapped to EORTC questionnaires QLQ-C30 and QLQ-OES18. QLQ—Quality of life questionnaire; EORTC—European Organisation for Research and Treatment of Cancer.

The exposure is nutritional problems that comprise of two components namely nutrition impact symptoms and weight loss. Nutrition impact symptoms: A group of symptoms that compromise oral intake, and in turn nutrition, in patients with advanced cancer owing to the cancer itself, from anti-cancer treatment or co-morbidities is defined as nutrition impact symptoms [13,14,15,16,17]. The most commonly known nutrition impact symptoms were matched to the symptoms from the QLQ-C30 and the QLQ-OES18 (Figure 1) which were categorized as ‘0–1 symptoms’ and ‘at least 2 symptoms’ to define the first part of the exposure for this study.

Weight loss: The second part of the exposure was weight loss calculated from weight data collected from the patients as self-reported measures using study specific questionnaires during the follow-up. Percentage weight loss was calculated as ((weight (kg) at 6 months after surgery−weight before surgery (kg))/average weight as an adult (kg)) × 100 and categorised as greater than or less than median weight loss to define clinically relevant weight loss. The median was used since it is less sensitive to outliers and skewness. 

Nutritional problems: To define the final exposure i.e., nutritional problems, the combined effect of NIS and WL was assessed based on their severity that yielded four exposure groups: (1) Low (0–1 symptoms and <median weight loss), (2) Moderate (0–1 symptom and >median weight loss), (3) Severe (at least 2 symptoms and <median weight loss), and (4) Very severe (at least 2 symptoms and >median weight loss). Since high BMI before surgery is a known risk factor for severe weight loss greater >15% after surgery, the analyses were stratified by low BMI <25 and high ≥25.

#### 2.2.2. Outcome

The study outcomes were global QOL, social and physical function from the QLQ-C30 module and overall 5-year survival. Mean HRQOL scores were obtained by transforming the responses from the QLQ-C30 to linear scale scores of 0 to 100 as per the EORTC scoring manual. Missing values were handled according to the EORTC guidelines [18]. Since there were only 3 missing in the variable ‘tumour stage’ and 2 missing in the variable ‘type of operation’, we conducted complete case analysis due to very few missing cases. Adjusted mean score differences (MD) for the global QOL, social and physical function were calculated from ANCOVA regression models as: adjusted mean score of the reference exposure group at 6 months–adjusted mean score of respective exposure group at 6 months. For example, the MD for global QOL for the ‘moderate nutritional problem’ group with low BMI was obtained by deducting their adjusted mean score from that of ‘low nutritional problem’ group (reference group) i.e., 70–73 giving an MD of −3.

### 2.3. Statistical Analysis

An ANCOVA model was used to compare the reference exposure group (low nutritional problems) with the other three groups for HRQOL presented as MD with 95% confidence intervals (CI). Statistical significance was defined at *p* < 0.05. The evidence based guidelines for cross sectional data was used to determine the clinical significance of the MD as trivial (no difference or circumstances unlikely to be of clinical relevance), small (subtle but nevertheless clinically relevant), medium (likely to be clinically relevant but to a lesser extent than a large MD) or large (unequivocal clinical relevance) for the subscales of global QOL, social and physical function from QLQ-C30. The guidelines were used to interpret the changes in means scores with small modifications to avoid overlap between sub-groups. For example, the cut-off MD values for the global QOL sub-scale are: trivial, 0–3; small, 4–9; medium, 10–14; and large, more than or equal to 15. Only medium and large differences were defined as clinically relevant. 

A Cox proportional hazards model was used to explore the overall 5-year survival among the exposure groups presented as hazard ratios (HR) with 95% CI. The ANCOVA and survival models were adjusted for age (continuous), sex (males/females), co-morbidities (Charlson score 0/1/>2), histology (adenocarcinoma and dysplasia/squamous-cell carcinoma), tumour stage (0–I/II/III/IV), tumour location (upper and middle/lower and cardia) type of operation (oesophageal resection/cardia resection/extended total gastrectomy/total gastrectomy and oesophageal resection), surgical complications (no/yes). All analyses were stratified for BMI low (<25) and high (≥25). 

## 3. Results

### 3.1. Patients

Of 616 patients (100%) who underwent surgery, 506 (82%) were alive 6 months after surgery. A total of 402 (79%) answered the HRQOL questionnaires, 385 (76%) answered the weight and height questionnaire and 358 (71%) patients answered both questionnaires. Among this cohort, the median age at surgery was 66 years, a higher proportion of patients were men (81%), medical co-morbidities were relatively low in a majority (82% of patients had a Charlson co-morbidity score ≤ 1). Majority of the tumours were adenocarcinomas (77%) and were of pathologic stage II (32%) and III (39%). Of 358 included patients, 162 (47%) had a low pre-operative BMI and 196 (55%) had a high BMI before surgery. The low and high BMI groups group had a median weight loss of 7 kg and 11 kg, respectively.

Co-morbidities were higher among the high BMI than the low BMI (13% vs 6%) at surgery. Only 28% of patients with low BMI had adenocarcinomas compared to 48% among high BMI (Table 1). A majority of patients experienced very severe nutritional problems, i.e., 41% among low BMI and 46% among high BMI. About 34% experienced severe problems in both BMI groups. Moderate problems existed in 12% of patients among low BMI and 7% among high BMI. Low problems were prevalent among 13% in both BMI categories equally. 

### 3.2. Health-Related Quality of Life 

**Figure 2:**
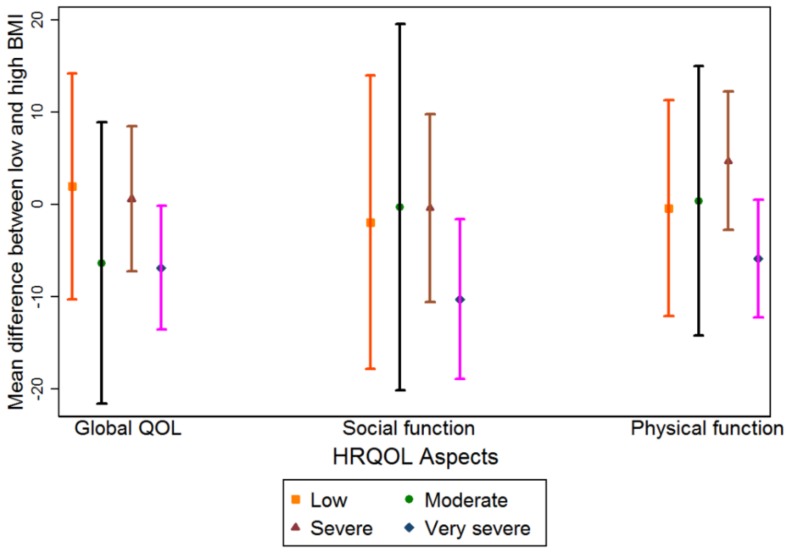
Chart showing mean score differences with 95% CI for global QOL, social and physical function among those with ‘low’, ‘moderate’, ‘severe’ and ‘very severe’ nutritional problems comparing low and high preoperative BMI groups. BMI—Body mass index; QOL—Quality of life; HRQOL—Health related quality of life; NIS—Nutrition impact symptoms; WL—Weight loss; CI—Confidence interval.

The mean scores of HRQOL for the reference exposure group (‘low’ nutritional problems) and the MD between the reference exposure group and the remaining three exposure groups are presented in Table 2. Those with ‘moderate’ nutritional problems showed no statistically or clinically significant differences in global QOL, social and physical function compared to the reference group in both categories of BMI. However, both the groups with ‘severe’ and ‘very severe’ nutritional problems reported clinically relevant and statistically significant mean score differences compared to the reference group for global QOL, social and physical function for both BMI categories (Table 2). For example, for global QOL a clinically large MD was observed for those with ‘severe’ nutritional problems with low BMI: MD −21 (95% CI: −32–−11) and high BMI: −20 (−29–−11). Likewise, for the ‘very severe’ nutritional problem group, the MD were −29 (95% CI: −39–−19) and −20 (95% CI: −29–−11) in the BMI categories low and high respectively. Considering MD between low and high BMI groups, only social function was statistically significantly different among those with ‘very severe’ nutritional problems with clinically small relevance MD −10 (95% CI: −19–−2) (Figure 2).

### 3.3. Overall 5 Year Survival 

**Figure 3:**
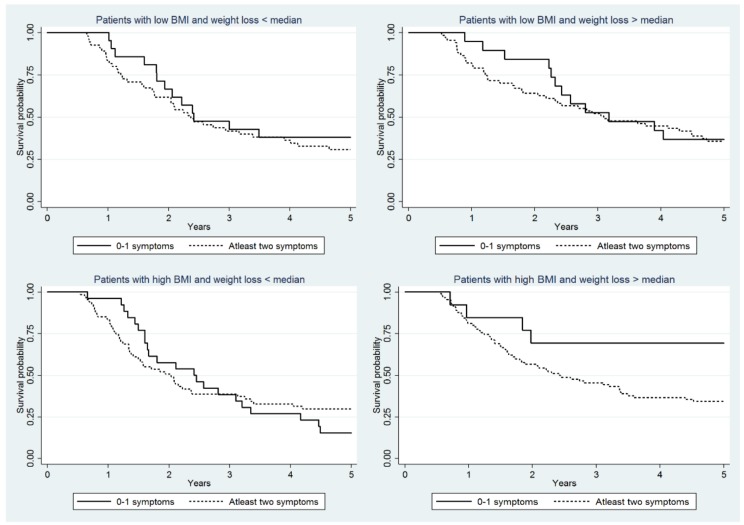
Panel diagrams of Kaplan Meier curves of overall 5-year survival of oesophageal cancer patients with at least 2 symptoms vs patients with 0–1 symptom as reference according to intensity of postoperative WL and preoperative BMI category. BMI—Body mass index; NIS—Nutrition Impact symptoms; WL—weight loss.

The survival probability for those with ‘at least 2 symptoms’ versus ‘0–1 symptom’ as the reference showed no statistically significant differences among low BMI and <median weight loss (HR 1.16 (95% CI 0.57–2.34) and >median weight loss (HR 1.92 95% CI 0.90–4.14) (Table 3, Figure 3). Likewise, among those with high BMI and <median weight loss there were no statistically significant differences in survival HR (0.92 95% CI 0.53–1.58) comparing at least 2 symptoms with 0–1 symptom. However, the survival among high BMI and >median weight loss was statistically significantly worse (HR 4.64 95% CI 1.38–15.56) for at least 2 symptoms compared to 0–1 symptom (Table 3; Figure 3). 

## 4. Discussion

This study indicates that presence of more symptoms affecting oral intake worsens global QOL, social and physical function at 6 months after operation for oesophageal cancer, irrespective of the degree of pre-operative BMI or post-operative weight loss. The presence of more symptoms is associated with poorer survival in those with high pre-operative BMI facing major post-operative weight loss. 

The population based design of the cohort that included almost all patients who underwent surgery during the study period in Sweden minimizes selection bias. A high response rate during the follow-up of patients in the cohort ensured a good categorisation for both parts of the exposure (nutrition impact symptoms and weight loss) and outcome (HRQOL) measures. The linkage of the cohort to the highly valid national Swedish registers provided a complete follow-up of the survival outcomes in the study and the ability to adjust for potential confounders, thereby displaying absence of confounding by these factors. A concern regarding baseline data for HRQOL in patients with cancer is that it is already affected by the cancer and hence not comparable to a healthy individual’s scores. The use of scores from the background population as proxy baseline scores overcomes this limitation. A weakness of the study is the small sample size within each exposure group. The evidence based guidelines to interpret the cut-off for the HRQOL scores is an advantage as they are accustomed for every sub-scale and thereby reduced overestimation or underestimation of scores. The data used in this study was collected during 2001–2005 and may be a limitation owing to changing clinical treatment regime. However, the results are independent of the type of treatment and thus may not be a big issue in the clinical interpretation of the results. In addition, tumour recurrence is associated with poor survival in patients operated for oesophageal cancer [19] the vast majority of patients have recurrence within the first 1–2 years of surgery [20,21,22] and may influence nutrition impact symptoms and HRQOL. In this cohort, however, a sub-analysis removing patients who died from oesophageal/gastric cancer at 9 months from surgery, i.e., 3 months after answering the 6-months questionnaire, showed similar results between the groups (data not shown) and thereby recurrence in unlikely to confound the results. 

Many population based studies have shown that the weight loss after surgery for oesophageal cancer is clinically significant [3,23,24]. Earlier studies have also shown that persisting gastrointestinal symptoms are a reason for concern after oesophagectomy [25,26]. This is the first study to show how these interact together to explain the deterioration in functional QOL and poor survival. A recent study from the same cohort showed that combined eating difficulties and clinically significant weight loss are a reason for clinically relevant deterioration in general HRQOL across a 10 year survival trajectory after surgery for oesophageal cancer [27]. The present study not only augments the earlier findings but also demonstrates them with relevance to other symptoms that hinder oral intake. Global QOL, social and physical functions are associated with poorer survival and the current findings indicate that more symptoms are an explanation for this association. Higher pre-operative BMI is recognized as a risk factor for malnutrition after oesophagectomy [28] and in this study is associated with poorer survival, strengthening these findings. 

Hindrance to oral nutritional intake may arise from complications of advanced cancer, anti-cancer treatment or medical co-morbidities. Oesophageal cancer is an aggressive form of cancer often diagnosed at a late stage and for which the treatment is extensive. Neo-adjuvant therapy followed by a major surgery resulting in anatomical changes may be an important reason for the occurrence of nutrition impact symptoms in this patient group. The surgical technique used may also be a key factor for the development of symptoms impacting oral intake [29]. 

The results of this study highlight the need for extensive nutrition support in patients who have undergone surgery for oesophageal cancer and the need for individualisation of the support based on factors such as presence of NIS, pre-operative BMI and post-operative WL. This warrants a systematic screening of NIS with HRQOL questionnaires; for example, the EORTC cancer cachexia module (QLQ-CAX25) consists of 25 questions related to cancer related malnutrition and WL. A guide to identifying and managing gastrointestinal symptoms in patients who underwent surgery for cancer has been suggested [30]. The present study also highlights the importance of understanding the nutrition symptom clusters after surgery for oesophageal cancer and their association with malnutrition to be able to decide on the sequential treatment and nutritional support specific to the symptoms also in the post-operative setting. At diagnosis or before the surgery, underweight patients receive nutritional support as they are screened by means of BMI. Those with higher BMI at diagnosis or surgery may be overlooked for the risk of malnutrition owing to the excessive stores of body fat, however it may be possible that a majority of them may have hidden sarcopenic obesity and thereby have a poorer survival when facing nutritional problems which is one of the key findings of this study. It may also be compounded greater when co-morbidities are present together with high BMI as Charlson co-morbidity index scores are shown to be directly associated with survival [31] and thereby represent a more vulnerable group who need heightened clinical care. 

## 5. Conclusions

The presence of more nutrition impact symptoms has clinically significant impact on global QOL, social and physical function at 6 months after surgery for oesophageal cancer. The overall 5-year survival among patients with more symptoms and weight loss is statistically significantly worse among those with a higher preoperative BMI. Identifying patients with severe nutrition impact symptoms with an appropriate screening tool is imperative, and patients who have higher BMI at diagnosis and surgery represent a group in need of greater clinical attention. 

## Figures and Tables

**Table 1:** Characteristics of patients who underwent surgery for oesophageal cancer in Sweden 2001–2005 and responded to questionnaires at 6 months after the surgery.

Characteristics	Total (*n* = 358)	Low BMI (*n* = 162)	High BMI (*n* = 196)
Sex			
Male	291 (81.3)	119 (33.2)	172 (48.0)
Female	67 (18.7)	43 (12.0)	24 (6.7)
Average age at operation *	66 (65–67)	66 (64–67)	65 (64–67)
Charlson co-morbidity score			
0	203 (56.7)	103 (28.8)	100 (27.9)
1	90 (25.1)	39 (10.9)	51 (14.3)
>2	65 (18.2)	20 (5.6)	45 (12.6)
Histology			
Adenocarcinoma and dysplasia	276 (77.1)	103 (28.8)	173 (48.3)
Squamous cell carcinoma	82 (22.9)	59 (16.5)	23 (6.4)
Tumour stage			
0–I	80 (22.6)	37 (10.5)	43 (12.2)
II	112 (31.6)	58 (16.4)	54 (15.3)
III	137 (38.7)	60 (17.0)	77 (21.8)
IV	25 (7.1)	4 (1.1)	21 (5.9)
Tumour location in oesophagus			
Upper and Middle	304 (84.9)	126 (35.2)	178 (49.7)
Lower and Cardia (Siewert II/III)	54 (15.1)	36 (10.1)	18 (5.0)
Type of operation			
Oesophageal resection	269 (75.6)	121 (34.0)	148 (41.6)
Cardia resection	16 (4.5)	3 (0.8)	13 (3.7)
Extended total gastrectomy	34 (9.6)	17 (4.8)	17 (4.8)
Total gastrectomy and oesophageal resection	37 (10.4)	20 (5.6)	17 (4.8)
Surgical complications			
No	243 (67.9)	115 (32.1)	128 (35.8)
Yes	115 (32.1)	47 (13.1)	68 (19.0)

Values are number of patients and percentages within brackets unless specified; * Average age in years (95% confidence interval); BMI–Body mass index before operation calculated as—(Weight (kg) before surgery/Height (m^2^)) and stratified as low (<25) and high (≥25); m^2^— Square meter.

**Table 2:** Health related quality of life scores of patients who underwent surgery for oesophageal cancer comparing the reference exposure group to the other three exposure groups stratified by preoperative BMI.

Nutritional Problems	Low BMI			High BMI		
	Global QOL MD 95% CI	Social Function MD 95% CI	Physical Function MD 95% CI	Global QOL MD 95% CI	Social Function MD 95% CI	Physical Function MD 95% CI
Low (Reference)	73 (63–84)	87 (73–100)	84 (74–94)	71 (62–81)	89 (76–101)	84 (75–94)
Moderate	−3 (−17–10)	−2 (−19–16)	−1 (−14–11)	5 (−9–19)	−3 (−22–15)	−2 (−16–12)
Severe	−21 (−32 to −11) *^††^	−23 (−37 to −9) *^††^	−14 (−24 to −4) *^†^	−20 (−29 to −11) *^††^	−24 (−37 to −12) *^††^	−19 (−28 to −10) *^†^
Very severe	−29 (−39 to −19) *^††^	-28 (−41 to −14) *^††^	−23 (−33 to −13) *^††^	−20(−29 to −11) *^††^	−19 (−31 to −7) *^††^	−17 (−26 to −9) *^†^

Low–0–1 symptom and < median weight loss; Moderate–0–1 symptom and > median weight loss; Severe–at least 2 symptoms and < median weight loss; Very severe–at least 2 symptoms and >median weight loss. * Statistically significant at *p* < 0.05; ^†^ Medium clinical significance as per evidence based interpretation guidelines compared to reference exposure group; ^††^ Large clinical significance as per evidence-based interpretation guidelines compared to reference exposure group; An ANCOVA model was used to compare the HRQOL scores of the reference group with the other three exposure groups. Values for reference group are mean scores with 95% CI, Values for the other three groups are mean score differences calculated from the ANCOVA models as adjusted mean score of respective exposure group at 6 months–adjusted mean score of reference exposure group at 6 months; BMI–Body mass index before operation calculated as—(Weight (kg) before surgery/Height (m^2^)) and stratified as <25 and ≥25; m^2^—Square meter; QOL—Quality of life; CI—Confidence interval; Symptom–Nutrition impact symptoms mapped from QLQ-C30 and the QLQ-OES18; Weight loss—Percentage weight loss calculated as ((weight (kg) at 6 months after surgery–weight before surgery (kg))/average weight as an adult (kg)) × 100 and stratified as < and > median weight loss.

**Table 3:** Overall 5-year survival of patients who underwent surgery for oesophageal cancer with at least 2 symptoms versus 0–1 symptom among < and > median weight loss and low and high preoperative BMI.

At Least 2 Symptoms Vs 0–1 Symptom	<Median Weight Loss HR (95% CI)	*p* Value	>Median Weight Loss HR (95% CI)	*p* Value
Low BMI	1.16 (0.57–2.34)	0.68	1.92 (0.90–4.14)	0.09
High BMI	0.92 (0.53–1.58)	0.75	4.64 (1.38–15.56) *	0.01

Cox proportional hazards model adjusting for age, sex, co-morbidities, histology, tumour stage, tumour location, type of operation, surgical complications. * Statistically significant; Symptom–Nutrition impact symptoms mapped from QLQ-C30 and the QLQ-OES18; HR—Hazard ratio; CI—Confidence interval. BMI—Body mass index before operation calculated as—(Weight (kg) before surgery/Height (m^2^)) and stratified as <25 and ≥25; m^2^—Square meter; QOL—Quality of life; Weight loss—Percentage weight loss calculated as ((weight (kg) at 6 months after surgery−weight before surgery (kg))/average weight as an adult (kg)) × 100 and stratified as < and > median weight loss.
